# Comparison of liver resection and radiofrequency ablation in long-term survival among patients with early-stage hepatocellular carcinoma: a meta-analysis of randomized trials and high-quality propensity score-matched studies

**DOI:** 10.1186/s12957-024-03330-8

**Published:** 2024-02-19

**Authors:** Lingbo Hu, Jiangying Lin, Aidong Wang, Xingpeng Shi, Yingli Qiao

**Affiliations:** 1 Department of Hepatopancreatobiliary Surgery, Taizhou Hospital of Zhejiang Province Affiliated to Wenzhou Medical University, Linhai, Zhejiang China; 2 Department of Hepatopancreatobiliary Surgery, Taizhou Enze Medical Center (Group), Enze Hospital, Taizhou, Zhejiang China; 3 Department of Blood Purification, Taizhou Hospital of Zhejiang Province Affiliated to Wenzhou Medical University, Linhai, Zhejiang China

**Keywords:** Radiofrequency ablation, Liver resection, Hepatocellular carcinoma, Early stage, Meta-analysis

## Abstract

**Background:**

Whether radiofrequency ablation (RFA) and liver resection (LR) are comparable treatments for early-stage hepatocellular carcinoma (HCC) is controversial. We conducted this study to provide ample clinical evidence for the argument.

**Methods:**

The PubMed, Embase, Web of Science, and Cochrane Library databases were systematically searched to identify randomized controlled trials (RCTs) and propensity score-matched (PSM) studies that compared long-term outcomes of both RFA and LR for patients with early-stage HCC. The hazard ratios (HRs) with 95% confidence intervals (95% CI) of overall survival (OS) and disease-free survival (DFS) were calculated.

**Results:**

Thirty-six studies consisting of six RCTs and 30 PSM studies were included in this study, and a total of 7384 patients were involved, with 3694 patients being treated with LR and 3690 patients with RFA. Meta-analysis showed that LR provided better OS and DFS than RFA (*HR*: 1.22, 95% *CI*: 1.13–1.31; *HR*: 1.56, 95% *CI*: 1.39–1.74, respectively). A sensitivity analysis indicated that the results were stable. For the subgroup of patients with BCLC 0 stage, RFA and LR resulted in similar OS and DFS. For the subgroup of patients with single tumor sizes less than 3 cm, RFA reached similar OS (*HR*: 1.19, 95% *CI*: 0.90–1.58) but worse DFS compared with LR (*HR*: 1.45, 95% *CI*: 1.11–1.90). For the subgroup of ablation margin larger than 0.5 cm, LR still resulted in better OS than RFA (*HR*: 1.29, 95% *CI*: 1.09–1.53); while the ablation margin was larger than 1 cm, both RFA and LR resulted in similar OS. The modality of RFA was also a factor that affected results. Subgroup analysis showed that patients receiving ultrasound-guided RFA had worse OS and DFS than LR (*HR*: 1.24, 95% *CI*: 1.14–1.36; *HR*: 1.44, 95% *CI*: 1.25–1.66, respectively).

**Conclusions:**

Meta-analysis showed that LR provided better OS and DFS for patients with early-stage HCC. However, RFA and LR had similar effects on long-term survival in patients with BCLC 0 stage HCC. RFA and LR probably had similar effects on OS in patients with solitary HCC less than 3 cm or when the ablation margin was larger than 1 cm which need more studies to confirm. The effects of different modalities of RFA on long-term survival are needed for further assessment.

**Supplementary Information:**

The online version contains supplementary material available at 10.1186/s12957-024-03330-8.

## Introduction

Owing to its noticeable incidence, hepatocellular carcinoma (HCC) has markedly attracted clinicians’ attention [1]. A remarkable number of early-stage HCC (ES-HCC) cases were detected because of the regular surveillance for HCC recommended by the guidelines in Western countries [2, 3]. At present, liver transplantation is an ideal treatment for ES-HCC, which could satisfy the Milan criteria with a high 5-year survival rate [4]. Nevertheless, the shortage of liver donation and the high cost of liver transplantation restrict its widespread utilization. Thus, liver resection is recommended by the European Association for the Study of the Liver and the American Association for the Study of Liver Diseases for ES-HCC [2, 3]. However, most patients who are eligible for resection are also candidates for thermal ablation. Radiofrequency ablation (RFA) is a less morbid procedure, and long-term outcomes may be similar to resection, particularly for tumors with a size of < 2 cm. Therefore, RFA has been particularly recommended to treat ES-HCC [5–8].

Many retrospective studies demonstrated that RFA and LR had similar survival benefits for ES-HCC patients [9–19]. However, this conclusion is controversial. A noticeable number of retrospective studies indicated that LR could prolong the overall survival (OS) and disease-free survival (DFS) for ES-HCC compared with RFA [20–24]. The benefit of RFA over LR for treating potentially resectable HCC has been studied in several RCTs conducted in China, Japan, and Hong Kong [25–30]. However, these studies had mixed results; some concluded that LR is superior, while others noted that both yielded similar outcomes. Besides, the criteria differentiating tumor characteristics were consistent among RCTs [31]. Hence, whether RFA can be the primary treatment for ES-HCC remains controversial.

Hence, we conducted the present meta-analysis of RCTs and high-quality propensity score-matched (PSM) studies to elucidate the comparative survival benefits and detrimental influences of LR versus RFA for ES-HCC.

## Methods

### Search strategy

The current meta-analysis was conducted according to the Preferred Reporting Items for Systematic Reviews and Meta-Analyses (PRISMA) statement [32]. Two scholars independently conducted a comprehensive systematic search on the PubMed, Web of Science, and Cochrane Library databases to retrieve relevant articles published until December 21, 2022. Disagreements were resolved through discussions. The keywords used in the search included “hepatocellular carcinoma,” “HCC,” “radiofrequency ablation,” “hepatectomy,” and “liver resection.” The details of the search strategy are summarized in Supplementary materials S1.

### Eligibility criteria

The inclusion criteria were as follows:

- (1) definitive diagnosis of ES-HCC described in the previously published guidelines.
- (2) satisfying the Milan criteria for ES-HCC cases.
- (3) RCTs and propensity score-matched (PSM) studies.
- (4) reporting at least one survival outcome.
- (5) the availability of full text of searched articles.
- (6) researches published in English.

The exclusion criteria were as follows:

- (1) other types of liver cancer, such as cholangiocarcinoma or metastasized liver cancer.
- (2) data extracted from national databases.
- (3) articles without outcomes of interest.
- (4) reviews, case reports, and meeting abstracts.

### Data collection and quality assessment

Two scholars independently retrieved data from the included studies. The following data were collected: the first author’s name, year of publication, country, study design, inclusion criteria, number of participants, characteristics of participants and tumors, hazard ratios (HRs) of OS and DFS, the incidence of morbidity, and the length of hospitalization. The two scholars also independently assessed the quality of eligible studies with the Cochrane risk-of-bias tool for RCTs [33] and the Newcastle–Ottawa scale (NOS) score for PSM studies. Further information regarding the complementary criteria is summarized in Table 1. Disagreements between the two scholars were resolved through discussion.

**Table 1:** Characteristics of included studies

Author	Region	Design	Inclusion criteria	Group	Modality of RFA	No. of patients	Age	Gender (M/F)	HBV/HCV	Child–Pugh A/B	AFP (ng/ml)	Tumor size (cm)	Solitary/multiple	100% AR/NAR	100% LH (Y/N)	Resection margin	Ablation margin	Follow-up (months)	Survival (median (95% *CI*))
Zhang	China	PSM	Single tumor; ≤ 3 cm	LR		67	57.51 ± 8.37	50/17	56/6	67/0	189.00 ± 568.99	24.67 ± 5.97*	67/0	AR	NR	> 1 cm		96ψ	mOS: not reached mRFS: 47 (42–NA)
2022				RFA	Ultrasound guided or laparoscopic	67	57.78 ± 10.97	49/18	56/7	67/0	258.39 ± 578.19	24.2 8 ± 5.73*	67/0				> 1 cm	96ψ	mOS: 95 (79–NA) mRFS: 33 (26–51)
Takayama	Japan	RCT	≤ 3 nodules; ≤ 3 cm	LR		150	68 (63–74)^ζ^	112/38	27/97	139/10	NR	1.8 (1.5–2.2)ζ	135/15	NR	NR	NA		5.04 (0.36–9.49)η,δ	mRFS: 3.46δ
2022				RFA	Ultrasound guided	151	69 (63–74)^ζ^	108/43	33/94	149/2	NR	1.8 (1.5–2.3)ζ	136/15				NA	4.99 (0.00–8.70)η,δ	mRFS: 3.04δ
Liu	China	PSM	≤ 3 nodules; ≤ 3 cm	LR		103	63 (55–71)^ζ^	76/27	48/43	102/1	5(> 400)	56 (> 20)*	94/9	NR	Y	NA		14.5 (9.9–57.7)ζ	mOS: 73.6 mRFS: 49.5
2022				RFA	Ultrasound guided	103	63 (54–70)^ζ^	75/28	54/42	102/1	9(> 400)	60 (> 20)*	89/14				NA	14.5 (9.9–57.7)ζ	mOS: 81 mRFS: 36.4
Ko	Korea	PSM	Single tumor; 1–3 cm	LR		23	NR	NR	NR	NR	NR	NR	23/0	NR	Y	NA		NR	NR
2022				RFA	laparoscopic	23	NR	NR	NR	NR	NR	NR	23/0				NA	NR	NR
Kim	Korea	PSM	Single tumor; ≤ 4 cm	LR		61	59.4ψ	43/18	43/3	59/2	304.6 ± 1215.3	2.29 ± 0.8	61/0	NR	Y	NA		NR	NR
2022				RFA	Ultrasound guided	61	62.2ψ	52/9	46/3	55/6	173.6 ± 765.6	2.2 ± 0.8	61/0				NA	NR	NR
Filippo	Italy	PSM	BCLC 0/A stage	LR		22	82.8 ± 3.2	13/9	13/2	19/3	NR	15 (> 20)*	20/2	NR	NR	NA		NR	NR
2022				RFA	Ultrasound guided or open or laparoscopic	22	82.2 ± 2.4	16/6	15/1	21/1	NR	15 (> 20)*	20/2				NA	NR	NR
Cheng	China	PSM	BCLC 0/A stage	LR		99	63.60 ± 9.86	82/17	82/12	83/2	47 (6.0–423.0)ζ	2.31 ± 1.93	96/3	NR	Y	NA		34 (1–175)η	NR
2022				RFA	Ultrasound or CT guided	31	65.48 ± 11.73	22/9	22/8	27/2	34 (3.5–242.5)ζ	1.14 ± 0.70	28/3				> 1 cm	34 (1–175)η	NR
Li	China	PSM	Single tumor; ≤ 2 cm	LR		59	61 (35–82)ζ	39/19	28/34	56/2	5 (> 200)	1.9 (1.0–2.0)ζ	58/0	NR	NR	NA		NR	NR
2021				RFA	NA	59	61 (34–80)ζ	39/19	23/27	57/1	12 (> 200)	1.8 (1.0–2.0)ζ	58/0				NA	NR	NR
Lee,D	Korea	PSM	Single tumor; ≤ 3 cm	LR		118	59.5 ± 8.7	91/27	90/10	118/0	90.2 ± 309.0	1.84 ± 0.56	118/0	NR	Y	NA		NR	NR
2021				RFA	Ultrasound guided	118	60.5 ± 10.3	88/30	84/12	118/0	67.6 ± 173.4	1.87 ± 0.51	118/0				Completed	NR	NR
Conticchio	France and Italy	PSM	BCLC 0/A stage	LR		136	74.7 (70–86.1)η	104/32	22/68	116/20	NR	24.5 (7–50)*η	120/16	NR	NR	NA		NR	NR
2021				RFA	Ultrasound guided or open or laparoscopic	136	75 (70–88)η	98/38	10/73	114/22	NR	25 (10–50)*η	117/19				NA	NR	NR
Bai 1	China	PSM	BCLC 0/A stage	LR		250	45 (> 60)	212/38	250/0	226/24	126 (< 400)	94 (≤ 3)	199/51	NR	NR	> 0.5 cm		60.5 (3.1–154.6)η	NR
2021				RFA	Ultrasound guided	250	57 (> 60)	202/48	250/0	222/28	144(< 400)	106 (≤ 3)	207/43				> 0.5 cm	58.7 (3.3–147.5)η	NR
Bai 2	China	PSM	BCLC 0/A stage	LR		423	98(> 60)	260/55	423/0	287/28	368 (< 400)	357 (≤ 3)	411/12	NR	NR	NA		60.5 (3.1–154.6)η	NR
2021				RFA	Ultrasound guided	423	101 (> 60)	264/51	423/0	285/30	367(< 400)	349 (≤ 3)	415/8				NA	58.7 (3.3–147.5)η	NR
Pan	China	PSM	BCLC 0/A stage	LR		118	53.0 (45.2–61.0)ζ	101/17	100/NR	NR	22.6 (3.94–218)ζ	2.50 (1.85–3.50)ζ	98/20	NR	NR	NA		26.22 (1.30–44.73)η	mOS: 25.6 mRFS: 22.0
2020				RFA	Ultrasound guided	236	56.0 (45.0–64.0)ζ	206/30	215/NR	NR	8.61 (3.12–165)^ζ^	2.55 (1.90–3.23)ζ	199/37				Completed	24.20 (0.97–44.73)η	mOS: 23.4 mRFS: 13.3
Oh	Korea	PSM	Multiple, BCLC 0/A stage	LR		31	56.0 (52.0–66.0)ζ	23/8	27/NR	31/0	12.7 (6.9–63.4)ζ	14 (≤ 2)	0/31	NR	NR	NA		5.8 (3.4–7.1)η	NR
2020				RFA	NA	31	57.0 (50.0–66.0)ζ	26/5	25/NR	31/0	16.1 (6.3–127.4)ζ	18 (≤ 2)	0/31				NA	5.8 (3.4–7.1)η	NR
Chong	China	PSM	BCLC 0/A stage	LR		59	57.7 ± 10.5	46/13	48/4	59/0	71 (4.0–436)ζ	2.0 (1.6–2.8)ζ	56/3	NR	Y	NA		NR	NR
2020				RFA	Ultrasound or CT guided or laparoscopic	59	59.3 ± 11.0	46/13	48/4	58/1	15 (4.0–305.0)ζ	2.3 (1.5–2.7)ζ	56/3				NA	NR	NR
Ye	China	PSM	Single tumor; 3–5 cm	LR		154	103 (> 60)	141/13	135/2	139/15	78 (< 20) 29 (≥ 400)	113 (3–4) 41 (4–5)	154/0	NR	NR			NR	NR
2019				RFA	Ultrasound guided	154	103 (> 60)	134/20	134/5	144/10	77 (< 20) 27 (≥ 400)	111 (3–4) 43 (4–5)	154/0			NA		NR	NR
Wang	China	PSM	Single tumor; ≤ 2 cm	LR		80	56 (41–62)	66/14	74/6	66/14	17 (3–378)	1.8 (1.5–2.0)	80/0	NR	NR		0.5–1.0 cm	27ψ	NR
2019				RFA	NA	80	52 (44–62)	64/16	74/6	62/18	34 (6–348)	1.7 (1.5–2.0)	80/0			NA		27ψ	NR
K im	Korea	PSM	Single tumor; ≤ 2 cm	LR		48	56.2 ± 8.9	38/10	36/5	48/0	137.1 ± 255.3	1.57 ± 0.30	48/0	NR	NR			59.1 ± 37.3	NR
2019				RFA	Ultrasound or CT guided	48	58.7 ± 9.8	35/13	34/8	48/0	146.2 ± 280.7	1.53 ± 0.32	48/0				> 0.5 cm	63.3 ± 30.4	NR
Di Sandro	Italy	PSM	BCLC 0/A stage	LR		91	65 (62–72)ζ	NR	15/58	NR	27 (≤ 5) 24 (5–22) 23 (> 22)	20 (19–28)*ζ	91/0	NR	NR	NA		33 (17–56)ζ	NR
2019				RFA	Percutaneous ablation	91	65 (56–76)ζ	NR	13/62	NR	26 (≤ 5) 26 (5–22) 26 (> 22)	20 (17–26)*ζ	91/0				NA	33 (17–56)ζ	NR
Min	Korea	PSM	Multiple, BCLC A stage	LR		20	NR	NR	NR	NR	NR	NR	0/20	NR	NR	NA		NR	NR
2019				RFA	Ultrasound or CT guided (*n* = 54) or intraoperative (*n* = 8)	20	NR	NR	NR	NR	NR	NR	0/20	NR	NR		> 0.5 cm	NR	NR
Lee, S	Korea	PSM	Single tumor; ≤ 3 cm; perivascular	LR		62	55.2 ± 8.6	NR	47/9	NR	28.8 (7.4–135.8)ζ	NR	62/0	NR	NR	NA		NR	NR
2018				RFA	Ultrasound guided	62	56.0 ± 9.7	NR	49/8	NR	15 (5.7–73.2)ζ	NR	62/0				> 0.5 cm	NR	NR
Lee,H	Korea	RCT (terminated)	Single tumor; 2–4 cm	LR		29	55.6 ± 7.9	23/6	20/3	29/0	1671.6 ± 5887.5	22 (≤ 3) 7 (3–4)	29/0	NR	NR	NA		NR	NR
2018				RFA	Ultrasound guided	34	56.1 ± 7.4	24/10	23/4	34/0	158.7 ± 286.9	26 (≤ 3) 8 (3–4)	34/0				0.5–1 cm	NR	NR
Kato	Japan	PSM	BCLC 0/A stage	LR		70	68 (39–79)^η^	55/15	NR	69/1	13.3 (1.4–2813.3)η	20 (9–30)*η	59/11	NAR	NR	NA		NR	mOS: 59.5 mRFS: 26.1
2018				RFA	Ultrasound or CT guided	70	70(27–85)η	53/17	NR	69/1	12.8 (2.0–4556.4)η	20 (6–30)*η	60/10				NA	NR	mOS: 45.4 mRFS: 16.1
Chong	China	PSM	BCLC 0/A stage	LR		121	59.5 ± 9.5	101/20	110/0	121/0	31 (6–357)	25 (20–36)*ζ	121/0	NR	NR	NA		NR	NR
2018				RFA	NA	121	62.0 ± 10.0	95/26	106/0	121/0	17 (6–129)	25 (20–35)*^ζ^	121/0				NA	NR	NR
Ng	China	RCT	BCLC 0/A stage	LR		109	55 (31–82)η	89/20	99/5	107/2	58 (1–4880)η	2.9 (1–5)η	99/10	NR	NR	NA		93ψ	mOS: 118.8 mRFS: 39.5
2017				RFA	Ultrasound guided	109	57 (23–78)η	86/23	95/0	104/5	63.5 (2–18 070)η	2.6 (1–5)^η^	90/19				> 1 cm	93^ψ^	mOS: 93.5 mRFS: 23.7
Song	China	PSM	Single tumor; ≤ 4 cm	LR		78	48 (44–57)ζ	70/8	73/NR	78/0	38.5 (6.9, 281.9)ζ	33 (≤ 2) 45 (2–4)	78/0	NR	Y	NA		31.2 (21.1–49.5)η	mOS: 75 (66.8–83.9) mRFS: 75 (26–51)
2016				RFA	Ultrasound guided	78	48 (43–58)ζ	70/8	77/NR	76/2	43.0 (6.0, 181.7)^ζ^	40 (≤ 2) 38 (2–4)	78/0				> cm	43ψ	mOS: 70 (62.9–77.9) mRFS: 75 (26–51)
Liu	China	PSM	Single tumor; ≤ 2 cm	LR		79	61 ± 13	55/24	46/31	NR	136 ± 233	NR	79/0	NR	NR	> 1 cm		44ψ	NR
2016				RFA	Ultrasound guided	79	63 ± 12	52/27	36/30	NR	127 ± 307	NR	79/0				NA		NR
He	China	PSM	BCLC 0/A stage	LR		150	51.2 ± 12.1	124/26	150/0	146/4	29 (200–400) 121 (≥ 400)	2.8 ± 1.0	138/12	NR	NR	NA		58.2ψ	NR
2016				RFA	Ultrasound guided	109	52.8 ± 12.9	96/13	109/0	105/4	31 (200–400) 78 (≥ 400)	2.6 ± 1.0	100/9				> 1 cm	42.0ψ	NR
Yune	Korea	PSM	BCLC 0/A stage	LR		17	60.2¶	14/3	9/1	16/1	281,800¶	2.2¶	NA	NAR	NR	NA		41^ψ^	NR
2015				RFA	Ultrasound guided or laparoscopic	17	64.1¶	11/6	11/2	16/1	79,500¶	1.8¶	NA				> 1 cm	26ψ	NR
Lee1	Korea	PSM	BCLC 0/A stage	LR		147	64 ± 10	110/37	69/40	147/0	443 ± 2036	126 (≤ 3) 21 (> 3)	115/32	NR	NR	NA		NR	NR
2015				RFA	NA	147	64 ± 11	101/46	57/53	147/0	297 ± 1415	115 (≤ 3) 32 (> 3)	121/26				NA	NR	NR
Lee2	Korea	PSM	BCLC 0/A stage	LR		48	62 ± 12	37/11	20/12	35/12	332 ± 951	38 (≤ 3) 10 (> 3)	41/7	NR	NR	NA		NR	NR
2015				RFA	NA	48	67 ± 12	32/16	11/16	32/15	526 ± 1517	35 (≤ 3) 13 (> 3)	38/10				NA	NR	NR
Kang	China	PSM	BCLC 0/A stage	LR		99	54 (31–74)η	77/22	83/8	95/4	15.2 (1.0–3412.2)η	2 (1.1–3.0)	99/0	NAR	NR	NA		NR	NR
2015				RFA	Ultrasound or CT guided	99	55 (32–80)η	77/22	83/8	95/4	25.6 (1.0–1873)η	1.9 (1.1–3.0)	99/0				> 0.5 cm	NR	NR
Jiang	China	PSM	Multiple, BCLC A stage	LR		140	53 ± 12	123/17	129/NR	139/1	91 (< 400)	2.4 ± 0.6	0/140	NR	NR	NA		NR	NR
2015				RFA	Percutaneous (*n* = 81), laparoscopic (*n* = 19), and open (*n* = 60)	140	55 ± 12	118/22	121/NR	135/5	105 (< 400)	2.3 ± 0.6	0/140				NA	NR	NR
Fang	China	RCT	BCLC 0/A stage	LR		60	53.5 ± 11.0	46/14	52/NR	43/17	50 (> 200)	22.8 ± 3.5*	49/11	NR	NR	96.7% completed		NR	NR
2014				RFA	Ultrasound or CT guided	60	51.4 ± 8.1	42/18	55/NR	32/23	52 (> 200)	22.1 ± 5.2*	41/19				95% completed	NR	NR
Pompili	Italy	PSM	Single tumor; ≤ 3 cm	LR		116	67 (41–83)η	87/29	11/78	NR	11 (1–9000)η	2.3 (0.8–3.0)η	116/0	NR	NR	NA		NR	NR
2013				RFA	NA	116	69 (38–85)η	92/24	17/78	NR	20 (2–1105)^η^	2.3 (1.3–3.0)^η^	116/0				NA	NR	NR
Wang	China	PSM	BCLC 0 stage	LR		52	35 (≤ 60)	38/14	34/14	NR	11 (> 200)	NR	52/0	NR	NR	NA		2.3 (1.5 > 3.7)ζ,δ	NR
2012				RFA	Ultrasound guided	52	29 (≤ 60)	35/17	32/18	NR	10 (> 200)	NR	52/0				NA	2.5 (1.4–4.1)ζ,δ	NR
Huang	China	RCT	BCLC 0/A stage	LR		115	55.91 ± 12.68	85/30	104/6	106/9	32(> 400)	NR	89/26	NR	NR	> 1 cm		3.87 (0.1–)η,δ	NR
2010				RFA	Ultrasound guided	115	56.57 ± 14.30	79/36	101/4	110/5	21 (> 400)	NR	84/31				0.5–1 cm	3.1 (0.5–5)η,δ	NR
Chen	China	RCT	Single tumor; ≤ 5 cm	LR		90	49.4 ± 10.9	75/15	NR	90/0	60 (< 200) 6 (200–399) 24 (≥ 400)	42 (≤ 3) 48 (> 3)	90/0	NR	NR	> 1 cm		NR	NR
2005				RFA	Ultrasound guided	71	51.9 ± 11.2	56/15	NR	71/0	40 (< 200) 8 (200–399) 23 (≥ 400)	37 (≤ 3) 34 (> 3)	71/0				NA	NR	NR

*PSM* propensity score match,*RCT* randomized controlled trial, *BCLC* Barcelona Clinic Liver Cancer, *LR* liver resection, *RFA* radiofrequency ablation, *NR* not reported, *M*, male, *F* female, *HBV* hepatitis virus, B; *HCV* hepatitis virus C; *AFP* alpha-fetoprotein, *AR*, anatomic resection, *NAR* nonanatomic resection, *LH* laparoscopic hepatectomy, *CI* confidence interval, *OS* overall survival, *RFS* recurrence-free survival. *The unit of this data is millimeter. ζData were presented as median (interquartile range). ηData were presented as median (range). ψData were presented as median. ¶Data were presented as mean. δThe unit of this data is year

### Study definition and the target outcomes

Solitary tumors with a size of less than 5 cm and maximally three nodules with a size of less than 3 cm were considered early-stage HCC [2]. Herein, OS and DFS were considered as primary time-to-event outcomes. Data from multivariate Cox proportional hazard models were used to compute HRs and 95% confidence intervals (CIs) to estimate OS and DFS. The approach introduced by Tierney et al. was utilized as an alternative for computing HRs from Kaplan–Meier curves in case of the absence of survival data, especially the absence of HRs or 95% CIs [34]. Major complications were defined as Clavien-Dindo grade III or above [35].

### Statistical analysis

An inverse variance model was utilized to analyze OS and DFS, particularly log-transformed HRs and 95% Cis. The Mantel–Haenszel method was utilized for calculating the odds ratios (OR) and 95% CI of dichotomous outcome variables. Heterogeneity was assessed using the *χ*^2^ method (*I*^2^ of 25% as low heterogeneity; 50% as moderate heterogeneity). The selection of the test model was based on the heterogeneity level with the random-effects model for *I*^2^ > 50% [36]. The robustness of the conclusion was assessed by the sensitivity analysis. A funnel plot was used to visually illustrate the publication bias through regressive approaches introduced by Egger and Begg. Meta-regression was carried out based on the published year, sample size, study design, region, and inclusion criteria. Subgroup analysis was conducted considering the tumor size and number (single tumor less than 2 cm or 3 cm or 5 cm), laparoscopic hepatectomy (LH), nonanatomic resection (NAR), anatomic resection (AR), modality of RFA, surgical margin, ablation margin, and the results of meta-regression. The level of statistical significance was set at *P* < 0.05. All the data analyses were performed with R (version 4.1.2).

## Results

### Study search and selection

**Fig. 1:**
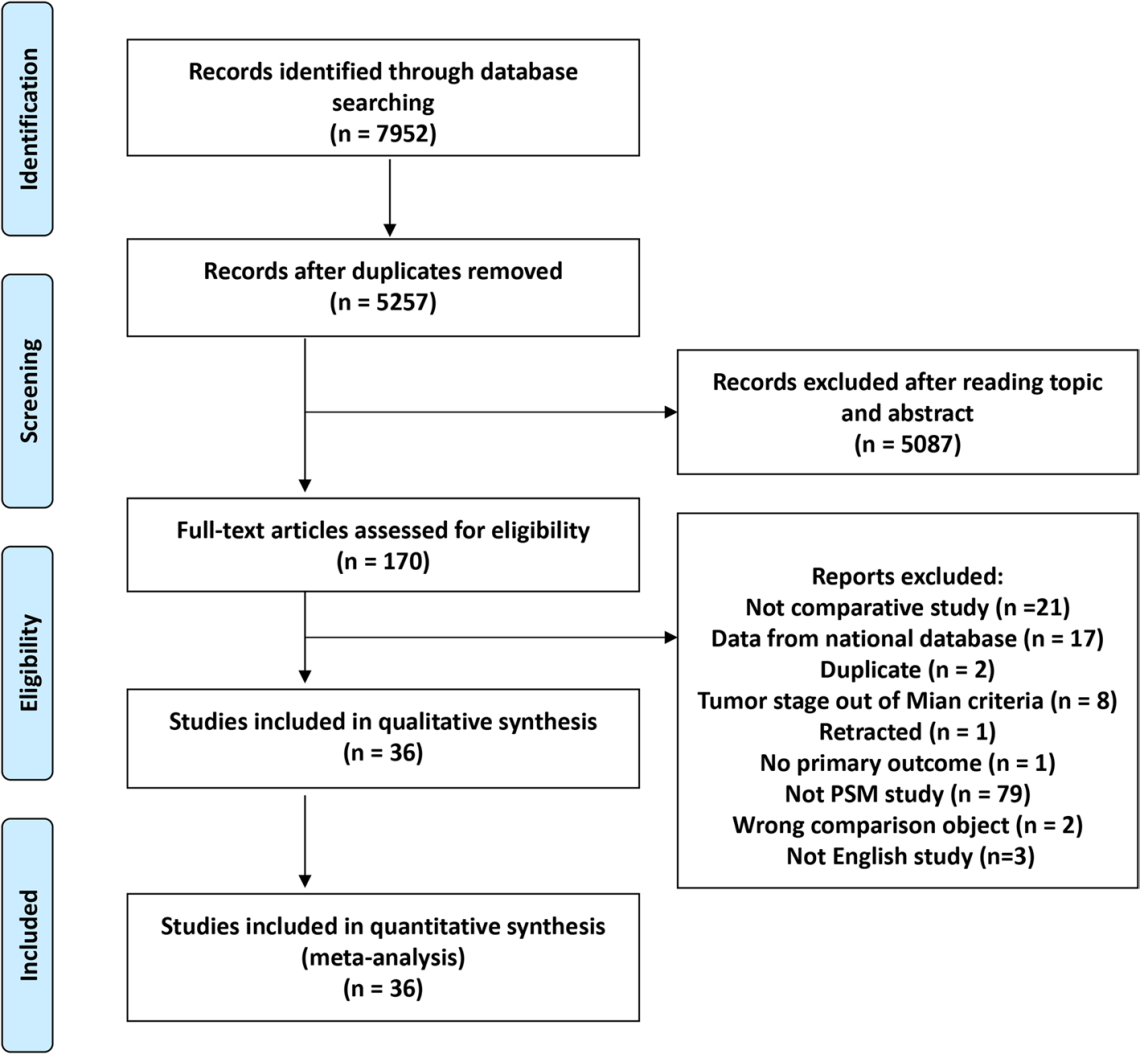
Flow chart of study selection

Database searching yielded a total of 5257 records, with 5087 excluded after reviewing the titles and abstracts (Fig. 1). For the remaining articles, 144 were further excluded because they did not meet the inclusion criteria. Finally, 36 studies were included in the meta-analysis (11, 14, 15, 24–30, 37–62).

### Study characteristics

The included 36 studies consisted of 6 RCTs and 30 PSM studies consisting of 38 datasets, involving a total of 7384 patients, with 3694 patients treated with LR and 3690 patients treated with RFA. These studies were conducted in China (*n* = 20), Korea (*n* = 10), Japan (*n* = 2), Italy (*n* = 3), and France and Italy (multicenter study) (*n* = 1). The quality of the included studies was assessed, and the results are shown in Supplementary materials S2 and S3.

Patient characteristics are shown in Table 1. Although all patients were eligible for BCLC 0/A, the inclusion criteria for tumor size and number varied among the included studies. Four studies involving 524 patients included BCLC 0 patients, and another four involving 638 patients included patients with single tumors ≤ 3 cm. Three studies compared RFA with NAR, and one compared RFA with AR. Six studies reported the comparison between RFA with laparoscopic hepatectomy (LH).

### OS, DFS, and recurrence

**Fig. 2:**
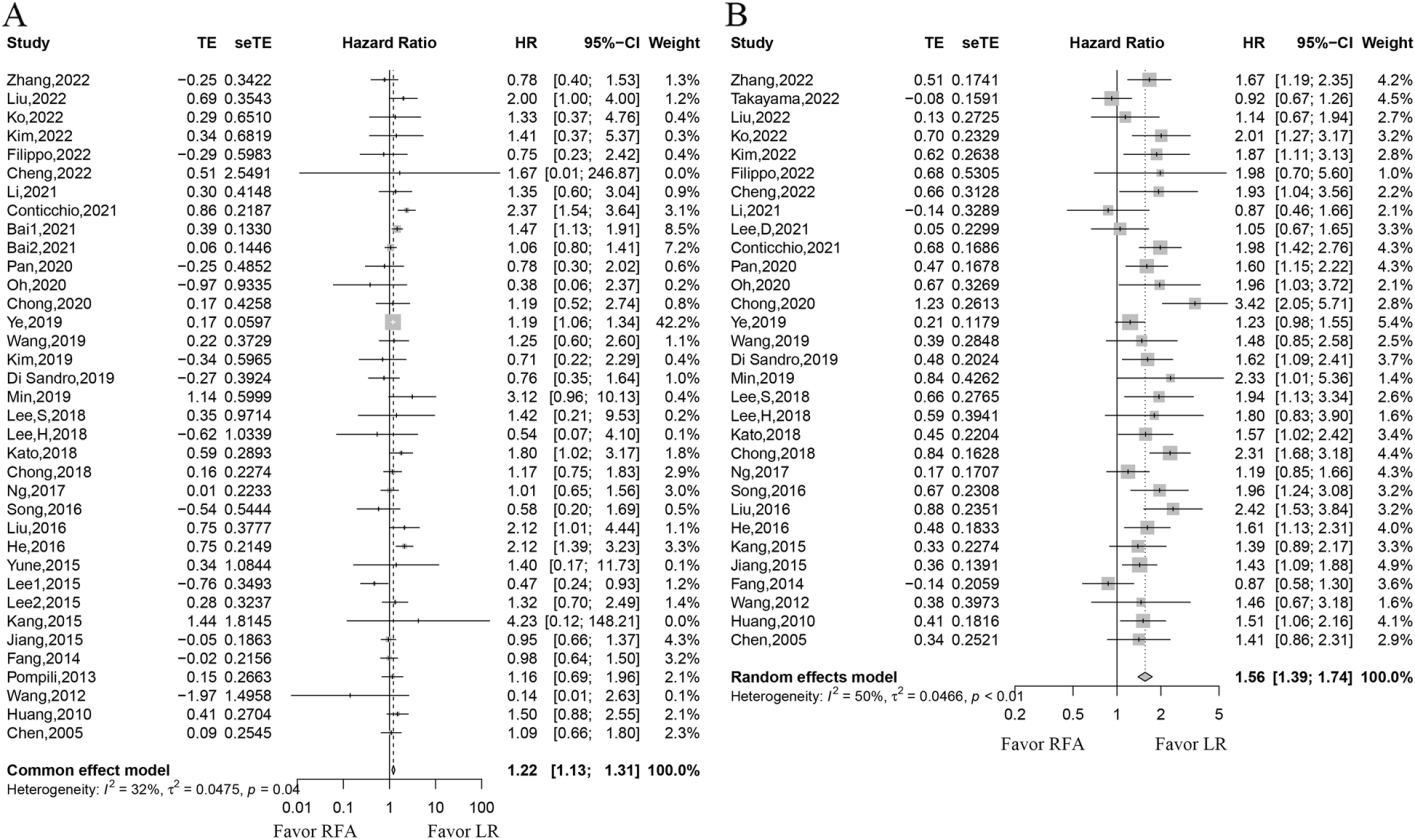
Forest plot for hazard ratios of overall survival (OS) and disease-free survival (DFS). **A** forest plot for OS. **B** Forest plot for DFS

The pooled analysis demonstrated that ES-HCC patients with a low level of heterogeneity undergoing RFA had significantly worse OS than those undergoing LR (*HR*, 1.22; 95% *CI*, 1.13–1.31; *P* < 0.01; *I*^2^ = 32%) (Fig. 2). In addition, ES-HCC patients with a moderate level of heterogeneity undergoing RFA had significantly worse DFS than those undergoing LR (*HR*, 1.56; 95% *CI*, 1.39–1.74; *P* < 0.01; *I*^2^ = 50%) (Fig. 2).

As shown in Supplementary S4, the survival and DFS rates were better in the LR group except for 1-year survival rates. A few studies reported that overall recurrence rate and 3- and 5-year recurrence rates were much higher in the RFA group (*OR*, 9.34; 95% *CI*, 1.54–56.59; *P* < 0.01; *I*^2^ = 91; *OR*, 4.78; 95% *CI*, 2.29–9.98; *P* < 0.01; *I*^2^ = 67%, respectively).

### Sensitivity analysis and publication bias

The sensitivity analysis showed that the results of OS and DFS were robust (Supplementary materials S5). Funnel plots of OS and DFS combined with Begg’s and Egger’s tests indicated no significant publication bias (Supplementary materials S6).

### Meta-regression and subgroup analysis

Meta-regression indicated that published year, sample size, study design, region, inclusion criteria, the proportion of solitary tumor, and modality of RFA significantly affected the results (Supplementary materials S7). Details of the subgroup analysis are shown in Table 2 and Supplementary material S8. The cumulative result of RCTs indicated no significant difference between RFA and LR in OS or DFS, while the cumulative result of PSM studies showed that LR is superior to RFA in both OS and DFS. For patients with BCLC 0 HCC, RFA and LR have comparable effects on OS and DFS. When the single tumor diameter increased to 3 cm, the OS between the RFA and LR groups was similar, while the DFS was better in the LR group. When the single tumor diameter increased to 5 cm, the OS and DFS were better in the LR group. Four studies explicitly reported resection marigin is > 1 cm, subgroup analysis showed similar OS between two groups but better DFS in the LR group. Nine studies and six studies explicitly reported ablation margins are > 0.5 cm and > 1 cm, respectively. Subgroup analysis showed that when ablation margin is > 0.5 cm, LR was superior to RFA on OS; however, the advantage of LR disappeared when ablation margin is larger than 1 cm. LR was better than RFA in DFS, whether the ablation margin was larger than 0.5 cm or 1 cm. For OS, the inconsistency was also found in other subgroups, including the subgroup of sample size < 100 or > 100, Asia or Europe, and published before or after 2015. Besides, subgroup analysis also showed that LR was superior to RFA on DFS. RFA can be performed with ultrasound, CT guidance, or open or laparoscopic surgery. The modalities of RFA were various among included studies. Subgroup analysis showed that patients receiving RFA performed with ultrasound guidance had worse OS and DFS compared with LR. After mixing a percentage of patients with CT-guided RFA into ultrasound-guided RFA, OS and DFS were similar between the two groups.

**Table 2:** Subgroup analysis of overall survival and disease-free survival

Subgroup	No. of datasets	HR	95% *CI*	*I*^2^	Model
OS					
Single tumor ≤ 2 cm	4	1.40	0.93–2.11	0%	Fixed
Single tumor ≤ 3 cm	8	1.19	0.90–1.58	0%	Fixed
Single tumor ≤ 5 cm	17	1.17	1.05–1.29	0%	Fixed
LH	6	1.33	0.87–2.03	0%	Fixed
NAR	3	1.81	1.05–3.10	0%	Fixed
PSM	31	1.24	1.14–1.34	38%	Fixed
RCT	5	1.09	0.86–1.37	0%	Fixed
Sample size < 100	23	1.14	0.95–1.36	0%	Fixed
Sample size > 100	13	1.26	1.03–1.53	63%	Random
Asia	32	1.2	1.11–1.30	22%	Fixed
Europe	4	1.24	0.70–2.20	69%	Random
China	21	1.21	1.11–1.31	21%	Fixed
Published after 2015	26	1.26	1.16–1.37	33%	Fixed
Published on or before 2015	10	1.03	0.86–1.24	14%	Fixed
Surgical margin > 1 cm	4	1.25	0.93–1.68	35%	Fixed
Ablation margin > 0.5 cm	9	1.29	1.09–1.53	0%	Fixed
Ablation margin > 1 cm	6	1.12	0.67–1.86	54%	Random
RFS					
Single tumor ≤ 2 cm	3	1.51	0.85–2.69	70%	Random
Single tumor ≤ 3 cm	8	1.45	1.11–1.90	66%	Random
Single tumor ≤ 5 cm	15	1.55	139–1.73	30%	Fixed
LH	7	1.78	1.32–2.39	59%	Random
NAR	2	1.48	1.09–2.02	0%	Fixed
PSM	25	1.64	1.51–1.78	35%	Fixed
RCT	6	1.15	0.98–1.35	38%	Fixed
Sample size < 100	20	1.68	1.50–1.88	33%	Fixed
Sample size > 100	11	1.42	1.20–1.67	62%	Random
Asia	28	1.54	1.36–1.73	52%	Random
Europe	3	1.83	1.43–2.34	0%	Fixed
China	19	1.54	1.34–1.77	54%	Random
Published after 2015	25	1.63	1.43–1.86	53%	Random
Published on or before 2015	6	1.52	1.41–1.64	3%	Fixed
Surgical margin > 1 cm	4	1.69	1.38–2.06	8%	Fixed
Ablation margin > 0.5 cm	7	1.42	1.22–1.66	0%	Fixed
Ablation margin > 1 cm	5	1.56	1.31–1.86	3%	Fixed

*HR* hazard ratio, *OS* overall survival, *LH* laparoscopic hepatectomy, *NAR* nonanatomic resection, *PSM* propensity score match, *RCT* randomized controlled trial, *DFS* disease-free survival

### Morbidity and hospital stay

**Fig. 3:**
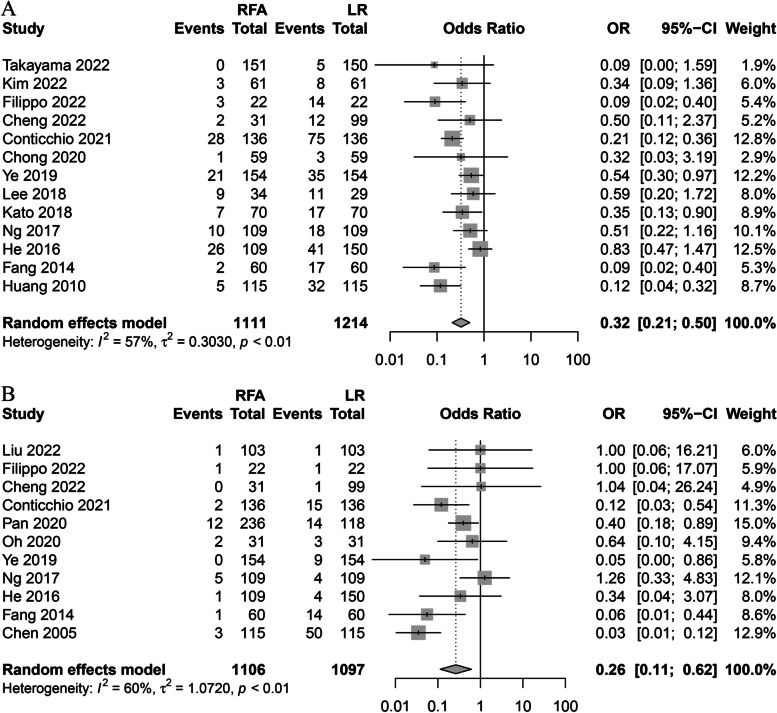
Forest plot for overall and major complications. **A** Forest plot for total complication. **B** Forest plot for major complication

**Fig. 4:**
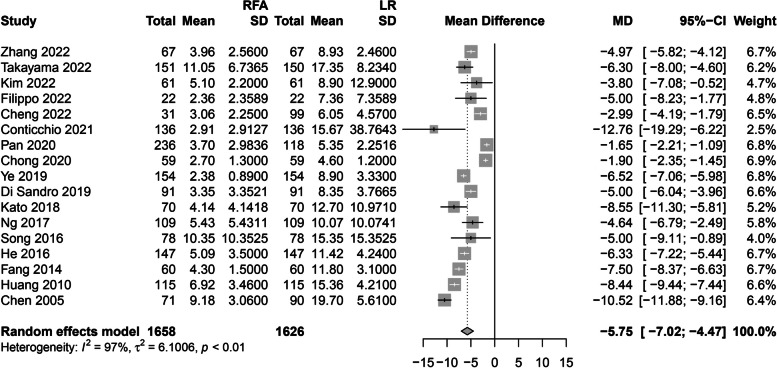
Forest plot for hospital stay

The incidences of postoperative overall and major complications were statistically lower in the RFA group than in the LR group (*OR*, 0.32; 95% *CI*, 0.21–0.50; *P* < 0.01; *I*^2^ = 57%; *OR*, 0.26; 95% *CI*, 0.11–0.62; *P* < 0. 01; *I*^2^ = 60%, respectively) (Fig. 3). The length of hospital stay was 5.75 days shorter in the RFA group than in the LR group (Fig. 4).

## Discussion

In this meta-analysis, meta-analysis showed that ES-HCC patients undergoing LR had better OS and DFS than those undergoing RFA. However, ES-HCC is a complex conceptual set of HCC with different diameters (0–5 cm) and different numbers (1–3 tumors). Additionally, details related to hepatectomy (including anatomic hepatectomy, laparoscopic hepatectomy, tumor resection margin) and radiofrequency ablation (including radiofrequency ablation guidance, ablation margin, and ablation equipment) will affect the survival of patients with HCC. Subgroup analysis showed that RFA and LR can provide similar OS and RFS for very early stage HCC (single tumor and the diameter less or equal to 2 cm). Additionally, when the tumor was single and less or equal to 3 cm, or the ablation margin wa larger than 1 cm, the OS provided by RFA and LR was similar, although the RFS was still better in LR. The incidence of postoperative complications was significantly lower, and hospitalization was significantly shorter among ES-HCC patients undergoing RFA.

The primary advantage of RFA over LR is less invasiveness. RFA causes minor damage to the surrounding healthy liver parenchyma, thus maximally preserving the liver remnant [37]. As a result, the complication rates were much lower, and the length of hospital stay was much shorter.

The main reason for the inferiority of RFA to LR in long-term survival is the higher local recurrence rate related to incomplete ablation [38]. The efficacy of RFA could be affected by several factors, including tumor number, tumor size, tumor location, RFA mode, RFA method, the level of regional medical care, and the experience of doctors [6, 39–42]. The insufficient ablation led to a high local recurrence rate [39]. On the other hand, LR could remove both the tumor and its micro neoplastic embolus by radically resecting primary cancer and adjacent liver parenchymal to guarantee a negative margin [43, 44]. In the subgroup analysis, we found that RFA can achieve similar OS to LR when the ablation margin was lager than 1 cm. Hence, the complete removal of the primary tumor and potential micrometastasis by LR might explain cothe superior long-term prognosis of early-stage HCC patients in the LR group.

Several meta-analyses have been available to compare the effects of RFA versus LR for HCC. Xu et al. performed a meta-analysis of five RCTs comparing survival outcomes of patients with small HCC who underwent LR or RFA [31]. RFA led to decreased overall survival compared with LR at 5 years, but the trial sequential analysis indicated that additional trials were necessary to confirm this conclusion. Additionally, time-to-event outcomes are most appropriately analyzed using HR [34]. Another recently published network meta-analysis by Zhang et al., which included RCTs and PSM studies, showed that LR is superior to RFA in OS and DFS [45]. The results are consistent with ours. However, their meta-analysis did not include one RCT and several PSM studies newly published in 2022. As far as we know, our meta-analysis is the most updated, with a maximum number of high-quality studies being included. More than 11,000 ES-HCC patients from 5 countries in the east and west were included to make the results more reliable and clinically meaningful. Moreover, sensitivity, subgroup, and meta-regression analyses provided ample evidence supporting our conclusion. The most important is that we focused on special subgroups which previous meta-analysis not did, including tumor number, tumor size, surgical margin, ablation margin, and even different guidance for RFA. Recently, a study based on Surveillance, Epidemiology, and End Results Program (SEER) database promped that RFA is an inferior option for solitary hepatocellular carcinoma ≤ 5 cm without cirrhosis [46]. This is an interesting and important finding because it lets us know that for HCC patients without cirrhosis, surgery is far a more suitable treatment than RFA. Because of insufficient data of liver cirrhosis in most of included studies and the proportion of liver cirrhosis of those studies reported, this data ranged from 2.2 to 94.1%, and we cannot confirm this view of the recent study. More well-designed studies are needed to verify this conclusion.

It should be noted that there are limitations for this study. First, we included both RCTs and PSM studies. Although the propensity score matching method could reduce baseline differences between groups, the deviations could not be eliminated compared with RCTs. Second, tumor heterogeneity could not be avoided. Although all the cases were ES-HCC, tumor number and size varied among patients in the included studies. Hence, we conducted a subgroup analysis; however, we found no significant difference between the two groups in OS among patients with a single tumor size of < 3 cm. However, extended subgroup analysis based on tumor number and tumor size is limited due to limited data. Third, the proportion of open LR or LH, anatomic or non-anatomic LR, are also inconsistent among included articles. Furthermore, with the development of RFA technology, various RFA techniques were used in different studies at different times. The influence of such heterogeneity has not been determined.

## Conclusion

In conclusion, this meta-analysis showed that LR provided better OS and DFS for patients with early-stage HCC. However, RFA and LR probably had similar effects on OS in patients with solitary HCC less than 3 cm or when the ablation margin was larger than 1 cm which need more studies to confirm. The effects of different modalities of RFA on long-term survival are needed for further assessment.

### Supplementary Information

**Additional file 1** PRISMA checklist

**Additional file 2** **Supplementary material:** **Supplementary material S1**: Search strategy. **Supplementary material S2** NOS score for PSM studies. **Supplementary material S3** Risk bias of RCTs. **Supplementary material S4** 1-,3-,and 5-year survival rate, disease-free survival rate, and recurrence rate. **Supplementary material S5** Forest plot for sensitivity analysis of overall survival and disease-free survival. **Supplementary material S6** Funnel plot for overall survival and disease-free survival. **Supplementary material S7** Meta-regression. OS, overall survival; DFS, disease-free survival; RFA, radiofrequency ablation. **Supplementary material S8** Subgroup analysis for OS and DFS based on modality of RFA

## Data Availability

The datasets used and/or analyzed during the current study are available from the corresponding author on reasonable request.
