# Supplementary material for: Comparison of liver resection and radiofrequency ablation in long-term survival among patients with early-stage hepatocellular carcinoma: a meta-analysis of randomized trials and high-quality propensity score-matched studies
Source: World J Surg Oncol. 2024 Feb 19;22:56. doi: 10.1186/s12957-024-03330-8 (PMC10875898; doi:10.1186/s12957-024-03330-8)

Supplementary material S1 Search strategy.

| Pubmed | #1**((((hepatectomy[MeSH Terms]) OR (liver resection)) OR (hepatic resection)) OR (hepatectomy)) OR (surgical resection)**  **#2(((radiofrequency catheter ablation[MeSH Terms])) OR (radiofrequency ablation)) OR (RFA)**  **#3 ((carcinoma, hepatocellular[MeSH Terms]) OR (hepatocellular carcinoma)) OR (HCC)**  **#4 #1 AND #2 AND #3** |
| --- | --- |
| Cochrane library | #1 MeSH descriptor:[Hepatectomy] explode all trees OR (liver resection) :ti,ab,kw OR (hepatic resection) :ti,ab,kw OR (hepatectomy) :ti,ab,kw OR (surgical resection) :ti,ab,kw  #2 MeSH descriptor:[Carcinoma, Hepatocellular] explode all trees OR (hepatocellular carcinoma):ti,ab,kw OR (HCC) :ti,ab,kw  #3 (radiofrequency ablation) :ti,ab,kw OR (RFA) :ti,ab,kw  #4 #1 AND #2 AND #3 |
| Embase | #1 'liver resection':ti,ab,kw OR 'hepatic resection':ti,ab,kw OR 'surgical resection':ti,ab,kw OR hepatectomy:ti,ab,kw OR 'hepatectomy'/exp  #2 'radiofrequency ablation':ti,ab,kw OR rfa:ti,ab,kw OR 'radiofrequency ablation'/exp  #3 '('liver cell carcinoma')/exp/mj OR 'hepatocellular carcinoma':ti,ab,kw OR hcc:ti,ab,kw  #4 #1 AND #2 AND #3 |
| Web of Science | **#1 (((ALL=(liver resection)) OR ALL=(hepatic resection)) OR ALL=(surgical resection)) OR ALL=(hepatectomy)**  **#2 (ALL=(radiofrequency ablation)) OR ALL=(RFA)**  **#3 (ALL=(hepatocellular carcinoma)) AND ALL=(HCC)**  **#4 #1 AND #2 AND #3** |

Supplementary material S2 NOS score for PSM studies

| Study | Selection | | | | Design | Analysis | Comparability | | Adequacy of follow up | Total score |
| --- | --- | --- | --- | --- | --- | --- | --- | --- | --- | --- |
|  | Representativeness of the exposed cohort | Selection of the non-exposed cohort | Ascertainment of exposure | Demonstration of outcome |  |  | Assessment of outcome | Follow-up was long enough |  |  |
| Zhang | * | * | * | * | * | * | * | * | * | 9 |
| Liu | * | * | * | * | * | * | * | * | * | 9 |
| Ko | * | * | * | * | * | * | * | * | * | 9 |
| Kim | * | * | * | * | * | * | * | * | * | 9 |
| Filippo | * | * | * | * | * | * | * | * |  | 8 |
| Cheng | * | * | * | * | * | * | * | * | * | 9 |
| Li | * | * | * | * | * | * | * | * | * | 9 |
| Lee,D | * | * | * | * | * | * | * | * |  | 8 |
| Conticchio | * | * | * | * | * | * | * | * | * | 9 |
| Bai | * | * | * | * | * | * | * | * | * | 9 |
| Pan | * | * | * | * | * | * | * | * |  | 8 |
| Oh | * | * | * | * | * | * | * | * | * | 9 |
| Chong | * | * | * | * | * | * | * | * | * | 9 |
| Ye | * | * | * | * | * | * | * | * | * | 9 |
| Wang | * | * | * | * | * | * | * | * | * | 9 |
| Kim | * | * | * | * | * | * | * | * | * | 9 |
| Di Sandro | * | * | * | * | * | * | * | * | * | 9 |
| Min | * | * | * | * | * | * | * | * | * | 9 |
| Lee,S | * | * | * | * | * | * | * | * | * | 9 |
| Kato | * | * | * | * | * | * | * | * | * | 9 |
| Chong | * | * | * | * | * | * | * | * | * | 9 |
| Song | * | * | * | * | * | * | * | * | * | 9 |
| Liu | * | * | * | * | * | * | * | * | * | 9 |
| He | * | * | * | * | * | * | * | * | * | 9 |
| Yune | * | * | * | * | * | * | * | * | * | 9 |
| Lee | * | * | * | * | * | * | * | * | * | 9 |
| Kang | * | * | * | * | * | * | * | * | * | 9 |
| Jiang | * | * | * | * | * | * | * | * | * | 9 |
| Pompili | * | * | * | * | * | * | * | * |  | 8 |
| Wang | * | * | * | * | * | * | * | * | * | 9 |
|  |  |  |  |  |  |  |  |  |  |  |

Supplementary material S3 Risk bias of RCTs

A, risk of bias summary; B, risk of bias graph.


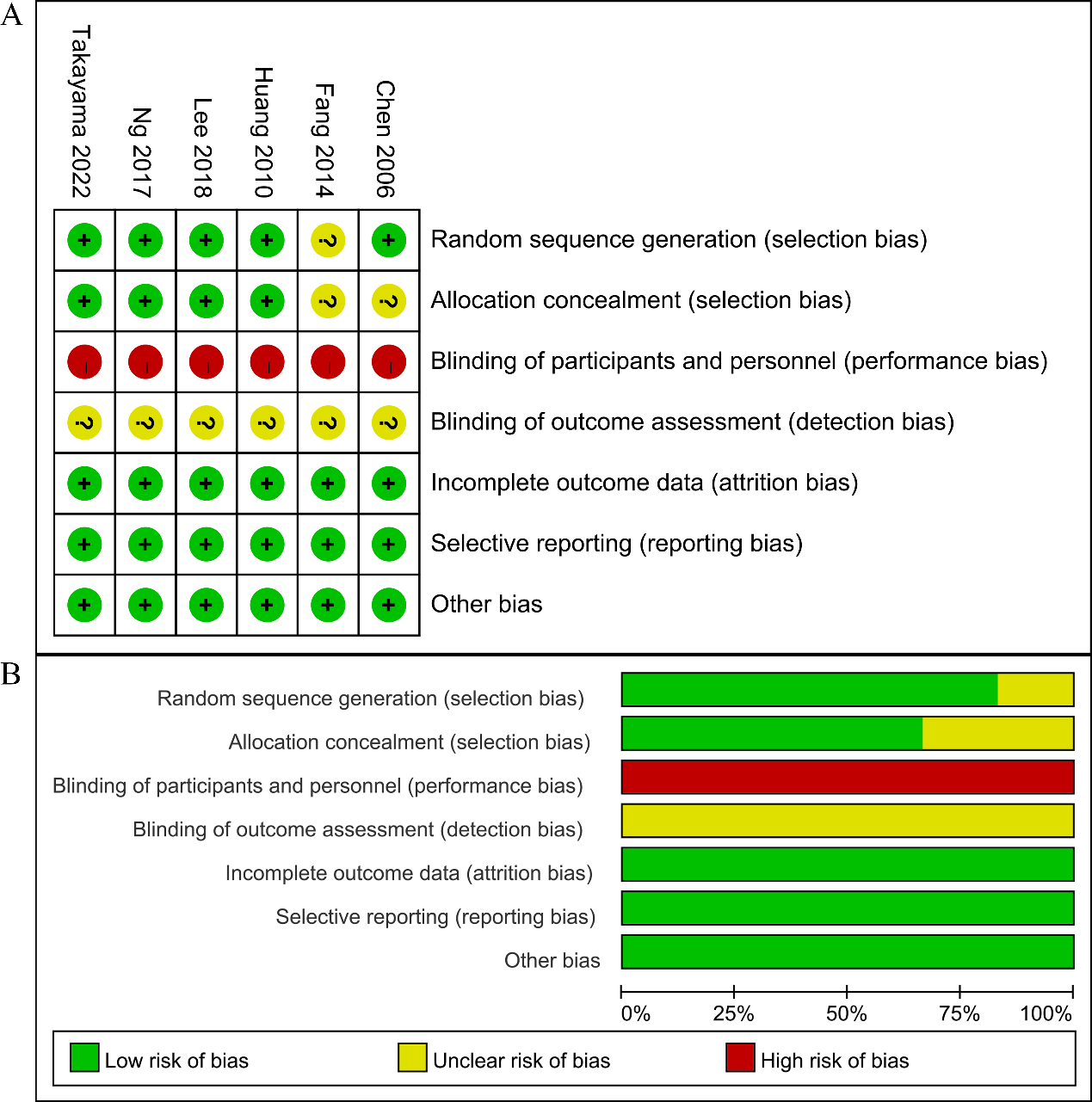


　　 Supplementary material S4 1-,3-,and 5-year survival rate, disease-free survival rate, and recurrence rate

| Outcomes | NO. of study | NO. of patient (RFA) | NO. of patient (LR) | OR | 95%CI | I^2^ | Model |
| --- | --- | --- | --- | --- | --- | --- | --- |
| 1-year survival rate | 25 | 2635 | 2645 | 1.00 | 0.78-128 | 12% | Fixed |
| 3-year survival rate | 31 | 3116 | 3121 | 0.68 | 0.54-0.86 | 55% | Random |
| 5-year survival rate | 26 | 2567 | 2671 | 0.58 | 0.47-0.72 | 60% | Random |
| 1-year disease-free survival rate | 22 | 1947 | 1957 | 0.48 | 0.37-0.61 | 51% | Random |
| 3-year disease-free survival rate | 27 | 2384 | 2388 | 0.51 | 0.45-0.57 | 49% | Fixed |
| 5-year disease-free survival rate | 23 | 1850 | 1953 | 0.43 | 0.34-0.55 | 62% | Random |
| 1-year recurrence rate | 7 | 1068 | 1068 | 0.60 | 0.30-1.20 | 80% | Random |
| 3-year recurrence rate | 7 | 1068 | 1068 | 9.34 | 1.54-56.59 | 91% | Random |
| 5-year recurrence rate | 5 | 935 | 935 | 4.78 | 2.29-9.98 | 67% | Random |
|  |  |  |  |  |  |  |  |

Supplementary material S5 Forest plot for sensitivity analysis of overall survival and disease-free survival.

A, forest plot for sensitivity analysis of overall survival. B, forest plot for sensitivity analysis of disease-free survival.


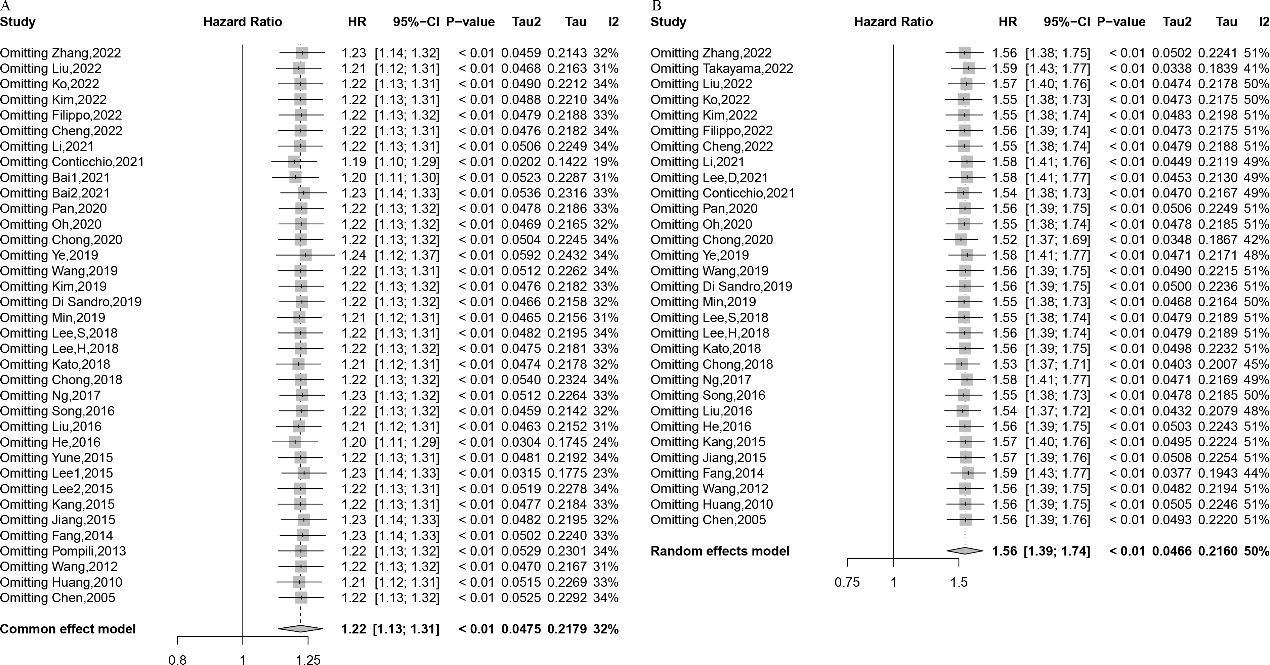


Supplementary material S6 Funnel plot for overall survival and disease-free survival.

A, funnel plot for overall survival. B, funnel plot for disease-free survival.


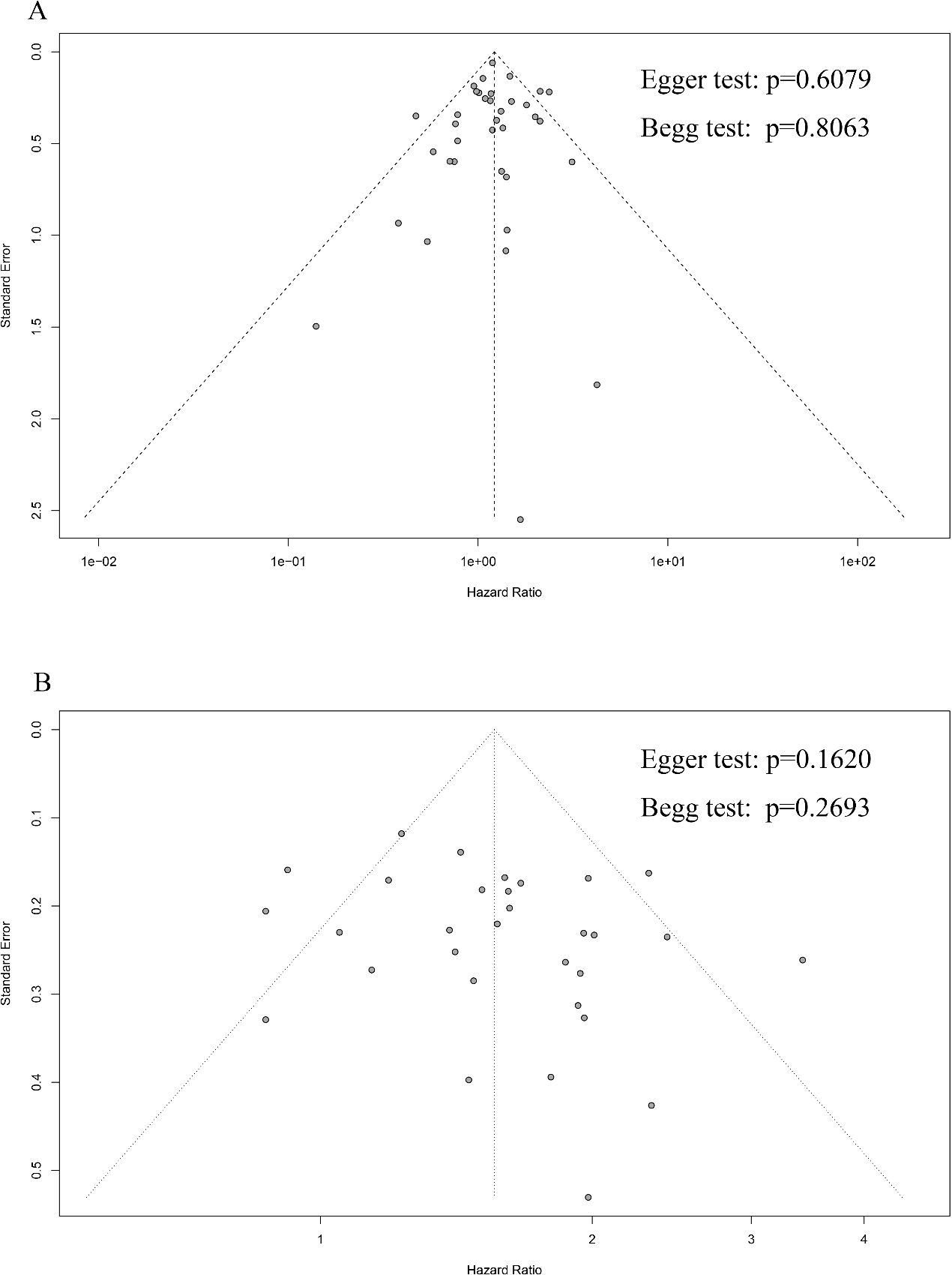


Supplementary material S7 Meta-regression

| Factors | Heterogeneity | | |
| --- | --- | --- | --- |
| OS | tau^2^ | I^2^ | P value |
| year | 0.0493 | 42.16% | 0.0343 |
| sample size | 0.05 | 42.02% | 0.0315 |
| design | 0.0491 | 41.85% | 0.0341 |
| region | 0.0463 | 40.85% | 0.0406 |
| inclusion criteria | 0.0715 | 41.97% | 0.0205 |
| the proportion of solitary tumor | 0 | 0% | 0.0049 |
| modality of RFA | 0.0311 | 31.54% | 0.0873 |
|  |  |  |  |
| DFS | tau^2^ | I^2^ | P value |
| year | 0.0494 | 52.12% | 0.0008 |
| sample size | 0.0432 | 48.58% | 0.0028 |
| design | 0.0254 | 35.70% | 0.0314 |
| region | 0.0468 | 50.59% | 0.0014 |
| inclusion criteria | 0.0594 | 55.20% | 0.0006 |
| the proportion of solitary tumor | 0.0499 | 51.72% | 0.0007 |
| modality of RFA | 0.0407 | 45.72% | 0.0162 |

OS, overall survival; DFS, disease-free survival; RFA, radiofrequency ablation.

Supplementary material S8 Subgroup analysis for OS and DFS based on modality of RFA

A, subgroup analysis for OS; B, subgroup analysis for DFS. US, ultrasound-guided; L, laparoscopic surgery; O, open surgery; CT, CT-guided; NR, not reported; PT, percutaneous; I, intraoperative.

A.


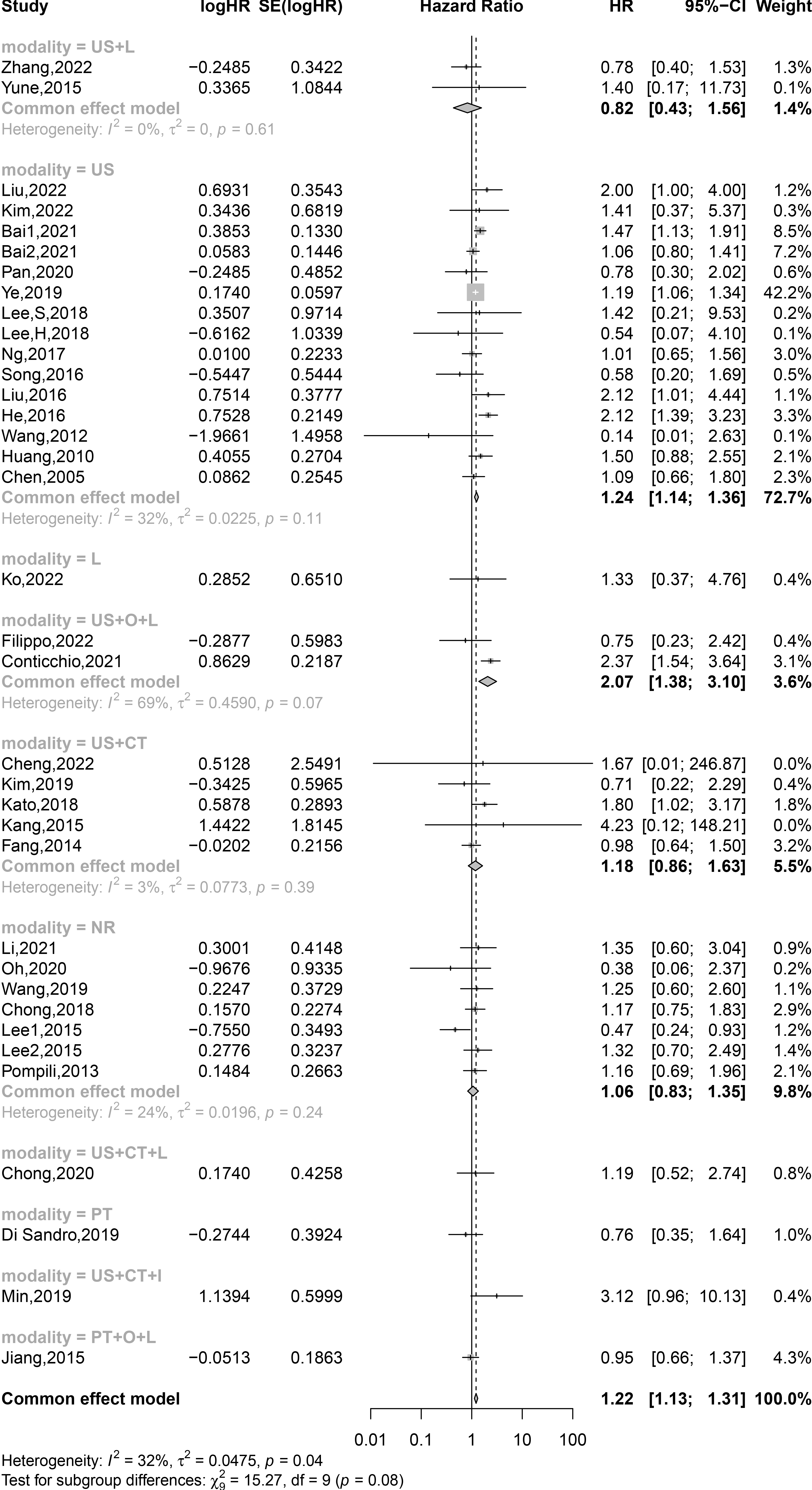


B.


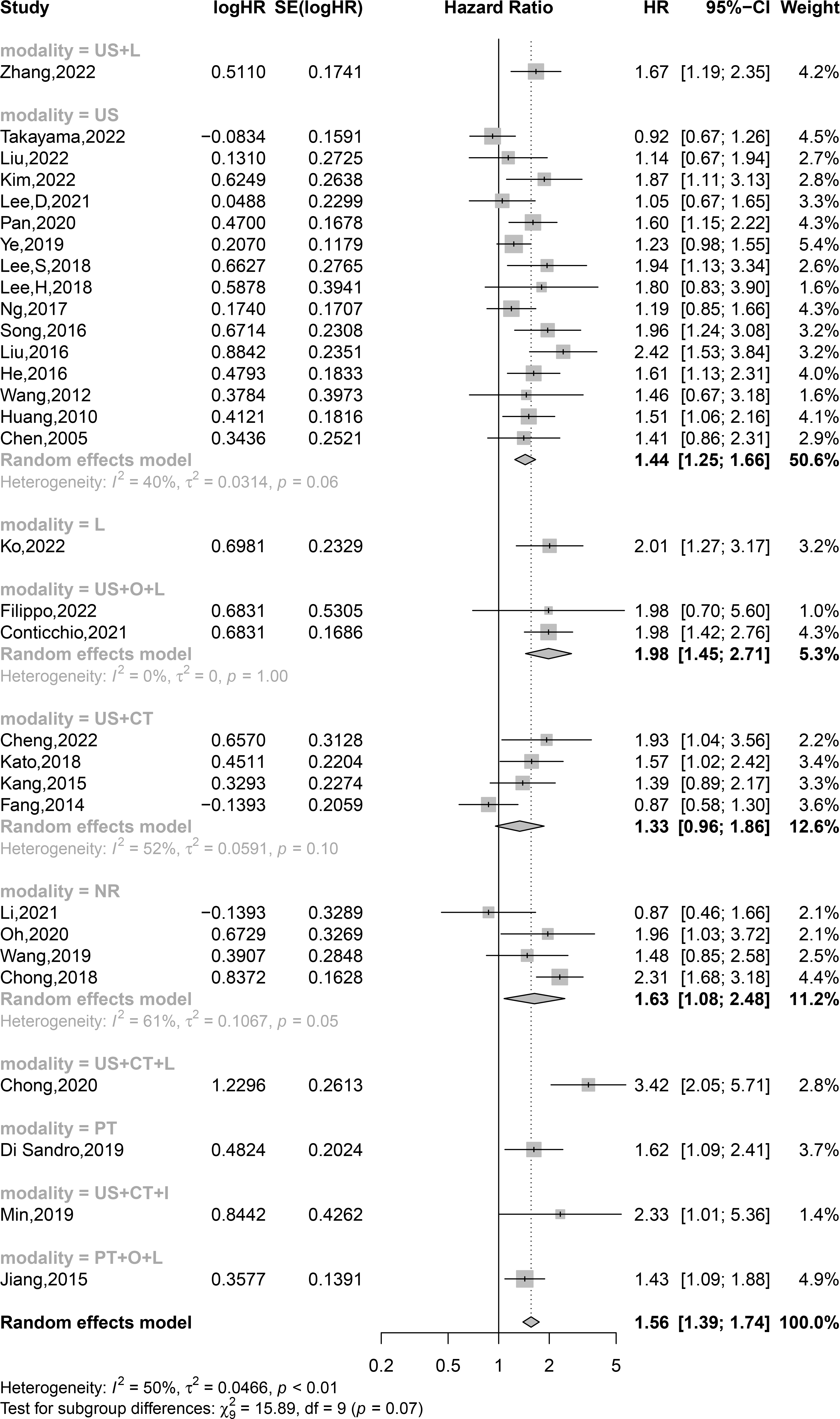

Supplement: Supplementary file 2 — Additional file 2 Supplementary material: Supplementary material S1: Search strategy. Supplementary material S2 NOS score for PSM studies. Supplementary material S3 Risk bias of RCTs. Supplementary material S4 1-,3-,and 5-year survival rate, disease-free survival rate, and recurrence rate. Supplementary material S5 Forest plot for sensitivity analysis of overall survival and disease-free survival. Supplementary material S6 Funnel plot for overall survival and disease-free survival. Supplementary material S7 Meta-regression. OS, overall survival; DFS, disease-free survival; RFA, radiofrequency ablation. Supplementary material S8 Subgroup analysis for OS and DFS based on modality of RFA [file 12957_2024_3330_MOESM2_ESM.docx]
